# Mechanical Characterization of Gres Porcelain and Low-Velocity Impact Numerical Modeling

**DOI:** 10.3390/ma11071082

**Published:** 2018-06-26

**Authors:** Cristiano Fragassa, Felipe Vannucchi de Camargo, Ana Pavlovic, Antonio Carlos de Figueiredo Silveira, Giangiacomo Minak, Carlos Pérez Bergmann

**Affiliations:** 1 Department of Industrial Engineering, Alma Mater Studiorum Università di Bologna, Viale del Risorgimento 2, 40136 Bologna, Italy; cristiano.fragassa@unibo.it (C.F.); ana.pavlovic@unibo.it (A.P.); giangiacomo.minak@unibo.it (G.M.); 2 School of Engineering, Federal University of Rio Grande do Sul, Avenida Osvaldo Aranha 99, 90035-190 Porto Alegre, Brazil; antonio.c.f.s@hotmail.com (A.C.d.F.S.); bergmann@ufrgs.br (C.P.B.)

**Keywords:** ceramic, annealing, drop-weight impact, finite element, LS-DYNA

## Abstract

The current investigation was conducted on gres porcelain stoneware, a robust, impermeable and aesthetically pleasing type of ceramic mainly used for flooring, characterizing its resistance to bending and low-velocity impact, both representative efforts to which flooring tiles are constantly subjected as a consequence of the fall of objects and microsubsidences. The mechanical characterization was made through experimental tests following an adapted low-velocity impact testing routine, and the model was by validated numerical simulation through the explicit code software LS-DYNA based on the Johnson–Holmquist constitutive material model. Specimens were tested before and after an annealing cycle industrially used to allow porcelain folding. The thermal treatment demonstrated to infer a decrease in mechanical resistance on the material, understood as a consequence of its elevated maximum temperature and fast cooling rate. The numerical model calibrated successfully allows predicting the behavior of gres porcelain before and after annealing against low-velocity impact.

## 1. Introduction

The class of ceramic materials is widely known in tiles production for being able to demonstrate elevated hardness, compressive strength and temperature endurance standards, being used in diverse industry segments and applications such as flooring, armor [1] and even spaceships [2]. Thus, ceramic tiles manufacturing represents an important industry segment with recent worldwide annual production indexes surpassing 13 billion square meters of tiles [3]. Given that ceramics are a tailor-made composition of different chemical elements to offer particular properties, there are various types and compositions specifically designed for each desired application.

Porcelain stoneware tiles, for instance, are greatly appreciated for flooring once they have good abrasion, chemical and frost resistance, enhanced aesthetics and present low water absorption of nearly 0.1% [4]. These features, apart from the material composition, are mainly due to the low-porosity of this type of ceramic, which is especially useful for flooring because it prevents the tiles from absorbing moisture from the slab, and at the same time assures high bulk density, tensile modulus and bending resistance.

Among other porcelains, gres is particularly appreciated for its complete impermeability and extreme resistance, where the aforementioned nomenclature gres stands for the vitrified, i.e., compact, form of the structure. Created in the 1970s [5], the floor gres was a pioneer on technical porcelain, patenting innovative production technologies such as the double loading and the vertical loading press, allowing to obtain advanced characteristics both technically and aesthetically without the need of vitreous enamel, which makes this material environmentally favorable and strongly considered within eco building guidelines.

**Figure 1:**
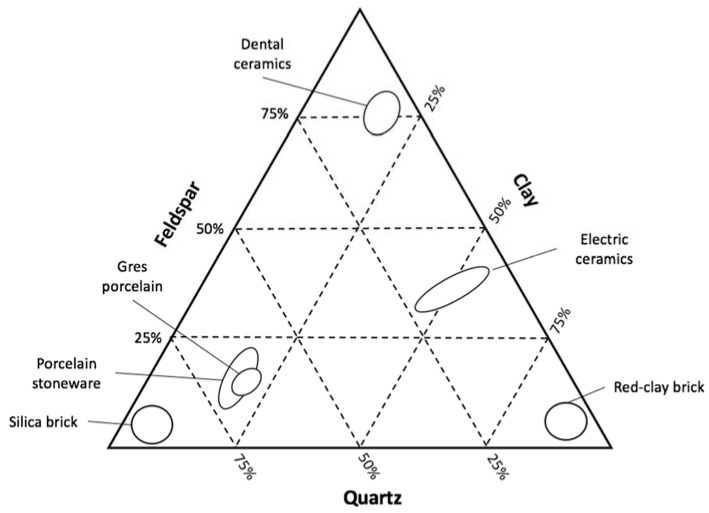
Graph of the main compositions of relevant types of ceramic materials built with data from Richerson et al. [8].

The basic composition of porcelain is made by quartz, feldspar and clay or kaolin. Quartz allows the composite to have high thermal and dimensional stability due to its elevated melting point; the glassy phase at low temperature of feldspar assists the sintering process and reduces the porosity at a very low level (less than 0.5% of open porosity and less than 10% of closed porosity); and clay is responsible for the plasticity and dry mechanical strength before firing, where it develops mullite that by its turn increases the bending strength of the material [6,7]. A general overview of some of the most relevant ceramic compositions can be seen in Figure 1.

Differently from metals, where plastic deformations are originated by the displacement of line defects within a plane of the crystalline structure followed by the reconstitution of the metallic bonds due to atomic electric charges, the deformation mechanisms of porcelain ceramic materials are particular, having in sight the predominance of directional covalent bonds [8]. The solicitation limit infers a terminal brittle fracture, independent of the monocrystalline or polycrystalline state of the material. On the other hand, polycrystalline ionic bonded ceramics are more brittle showing fractures on the grain boundaries [8].

The main issues that influence the mechanical resistance of porcelain are comprised within its own structure, i.e., porosity, superficial cracks or inclusions of grains and impurities [8]. Pores not only decrease the transversal section area thus diminishing the material strength, as they are stress concentration regions from where the cracks appear and through where they propagate. For this reason, porcelain ceramics (especially gres stoneware) are desirable materials, given their low intrinsic porosity and consequent enhanced strength.

Recent studies have been conducted to study aspects of gres porcelain. The confrontation between constituent elements and mechanical performance was explored by Dal Bó et al. [9,10], showing that through chemical tempering with potassium nitrate, the flexural strength of gres can be improved up to 74% allowing a 15% thickness reduction of tiles still obtaining superior resistance thresholds; and by Leonelli et al. [11], who assessed the flexural strength enhancement of specimens with mullite and kyanite as a function of a more reactive crystal interface. Delavi et al. [12] examined the influence of residual stress on gres porcelain submitted to cutting tests, where it was concluded that the greater is the stress level, the worse is the cutting behavior. Fernandes et al. [13] investigated tribological aspects of gres porcelain tiles, showing that a roughness decrease is more efficiently obtained by coarse grain abrasives, while fine abrasives are mainly responsible for the attainment of a glossy and polished visual aspect. Mahdi [14] proved that, by a controlled sintering temperature increase, the bending strength and micro-hardness of gres porcelain can be improved up to 13% and 7%, respectively.

In different and particular aspects, gres ceramics have also been studied for the novel usage on thermal diffuser panels on heat exchangers [15] (taking advantage of the superior mechanical properties, oxidation resistance and low porosity of this material), innovative anti-slip glazes [16], and for wastewater treatment [17,18,19] by photo degrading harmful disposed chemical substances that used to reach groundwater, surface water and even drinking water.

However, it is vital to highlight that the scientific production focused on subjects regarding porcelain tiles has been rare in the last decades, especially on the mechanical assessment of these materials, which does not reflect the significant technological advances, innovative material compositions, applicability and manufacturing breakthroughs achieved in this sector [6]. Even with comprehensive reviews mainly based on technical standard regulations [7], studies such as the one from Fragassa et al. [20] explicate an example of category of specimens of bent tiles built through novel manufacturing techniques that are not foreseen by standards, where adaptations of regulations and the proposition of new ones are made necessary.

Supported by the aforementioned statements, the scope of this work was based on the characterization of the scarcely studied, yet widely used gres porcelain. A further evaluation of its behavior was conducted on specimens submitted to a novel annealing routine applied for the industrial production of folded porcelain tiles [20], desirable for skirting boards, stairs, swimming pools and other constructions that involve the orthogonal assembly of tiles, providing more aesthetically pleasing and easy-to-clean aspects. Although advantageous in many facets, gres is also brittle and weak in tension [21], making a proper knowledge of its performance limitations important to avoid poor-designed tiles and end products.

Aiming at exploring important mechanical parameters of gres stoneware tiles, the present research evaluated the behavior of specimens before and after annealing to bending and low-velocity impact tests, the most important parameters to be properly known for a ceramic that is mainly applied for flooring as a consequence of microsubsidences and the fall of objects, respectively (particularly for impact, once localized contact with hard bodies is a known strength degradation mechanism of gres porcelain [22]). An experimental test routine based on technical standards was proposed to compensate for the lack of a specific one that allows the assessment of the force–time impact curve of the material to properly understand its mechanics and propel research on this field.

Furthermore, to analyze the impact response of the material, numerical simulations were conducted making use of the Johnson–Holmquist constitutive material model in the explicit-code software LS-DYNA (LSTC, Livermore, CA, USA). The software calibration achieved and hereby explained allows a practical prediction of dynamic solicitations at low energy levels on gres porcelain before and after the considered annealing.

## 2. Materials and Methods

### 2.1. Material

**Figure 2:**
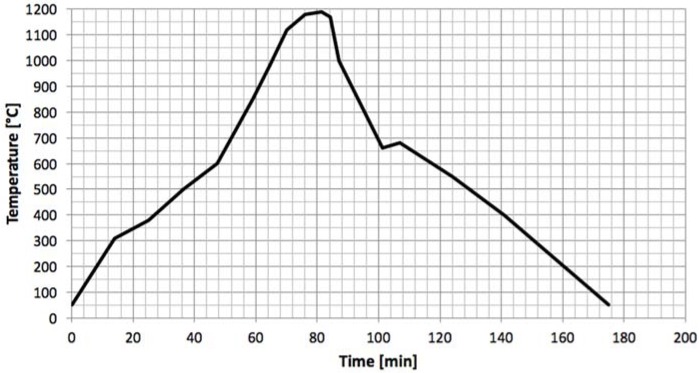
Annealing thermal cycle.

The gres porcelain considered was the FloorTech 1.0 manufactured by Florim Ceramiche S.p.A. (Fiorano Modenese, MO, Italy), being majorly composed of silica (SiO_2_, 69%), alumina (Al_2_O_3_, 21%) and small quantities of the oxides TiO_2_, Fe_2_O_3_, MgO, CaO, Na_2_O and K_2_O, according to the gres characterization indicated by other studies [4,20]. The resulting density was 2200 g/mm^3^. Specimens were separated into two groups: the regular and the annealed ones. Annealing is a thermal treatment destined to eliminate internal stress concentrations derivate from the manufacturing process and to promote/complete chemical reactions towards a thermodynamic equilibrium; its influence was studied in the present work to understand how the properties of gres vary depending on annealing. The thermal cycle (Figure 2) lasted for 3 h, where the ascending ramp rises until 1200 °C under a 15 °C/min rate, and then descends again to room temperature on a slower rate of 12 °C/min.

### 2.2. Experiment

**Figure 3:**
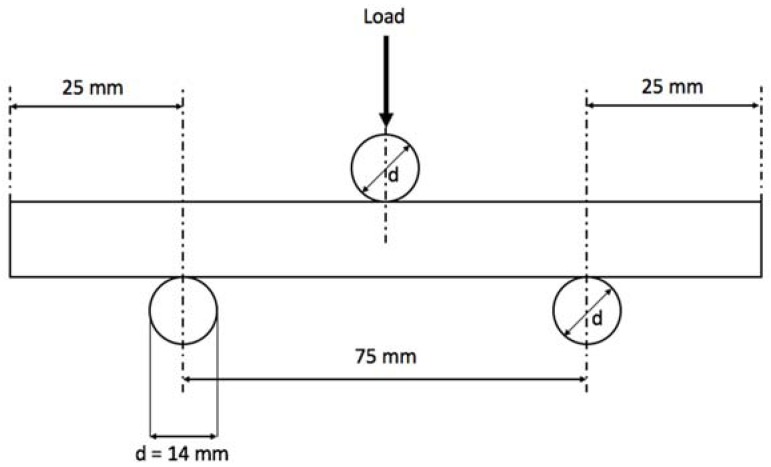
Bending test layout accounting for distances, loading direction and rod diameter (d).

As a common practice for fragile materials, where tension tests become difficult due to load alignment and sample clamping, bending tests are regularly applied to determine the transversal breaking strength. Gres porcelain coupons were cut into the dimensions 100 mm × 20 mm × 9.5 mm and submitted to three-point bending tests as indicated by the regulation ISO 10545-4 [23], which defines the standard process of a force applied at a specific rate in the center of the ceramic tile for determination of modulus of rupture and breaking strength of the material. The specimen was set to a 0.005 mm/s actuator displacement on a universal hydraulic testing machine where the rods were 14 mm in diameter and the distance between the lower rods was 50 mm, as illustrated by Figure 3. A total of 14 coupons were tested to assess statistical significance: 7 for regular and 7 for annealed ceramics.

The evaluation of the impact performance of ceramics is supposed to be done as indicated by the technical standard ISO 10545-5 [24]. It describes a test procedure in which a dropped weight falls from a known height in a way that the objective is to measure the rebound height of the impactor to define a dimensionless coefficient of restitution by dividing the first by the latter, without any mention to force or stress measurements. Other regulations intend to describe the superficial mechanical behavior of ceramics by merely analyzing its indentations in the case of ASTM C1327 [25] or by abrasion in the case of ASTM C1243 [26] and ISO 10545-6 [27]. However, drop-weight impact tests are very common for other categories of composite materials, demonstrating the validity of this analysis [20].

Given that these are the only standardized procedures to qualify the impact response of ceramic materials available in the literature, the present work proposes a more practical approach based on force–time data acquisition to understand the minimum impact capable of inferring a crack on the specimen, as well as to study the force–time curves that characterize the gres porcelain tested. It is possible to identify the initiation and development of damage during impact with these history curves.

**Figure 4:**
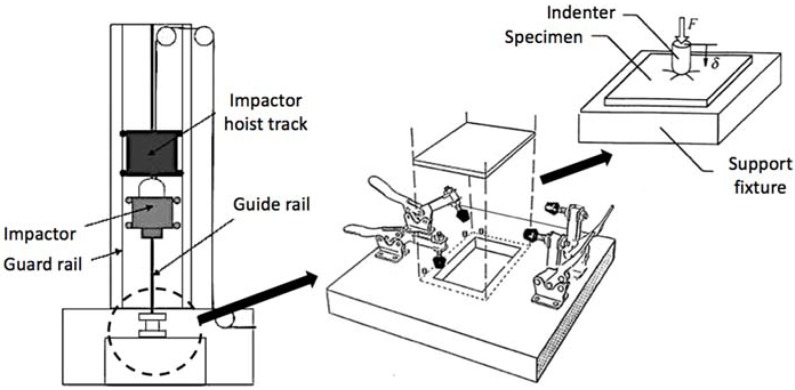
Drop-weight impact tower scheme.

The procedure proposed, based on an adaptation of the technical standard for fiber reinforced composite materials ASTM D7136 [28], was also made on a drop-weight tower testing machine and tried to compensate for the lack of literature guidelines on how to collect impact-related information from ceramic materials and at the same time promote the solid mechanics research of porcelain that are insufficiently studied [6]. Reducing the complexity of manufacturing tiles attached over concrete blocks as advised by ISO 10545-5, more functional coupons [28] were manufactured having as dimensions 150 mm × 100 mm × 10 mm, in which the displacement of the central portion of the specimens is allowed in the impact direction for a proper analysis of the energy absorption behavior of the tile itself, which is no longer limited by the effect of the adjacent concrete. A graphical representation of the drop-weight test tower used is depicted in Figure 4.

The test is based on the fall of a hemispheric steel impactor (of 12.5 mm in diameter) of known hardness (60 HRC) aided by guiding columns and magnets to hit exactly the center of the specimen. Given that the impactor weight is known (1.25 kg), the friction with the guiding columns can be neglected and the fall height is set, it is possible to accurately know the impact energy through basic physics. However, a velocity optical laser sensor is used to compute the impact velocity (through which all other data can be deduced using Newtonian physics). The rebound period (or maximum height) between two consecutive impacts can also be calculated to define the energy that has been dissipated by the shock.

The guiding column was positioned in a way that the specimen is located in the center of the support, which presents four mechanical clamps to fix the coupon during the impact experiment. The impactor was held at a pre-determined height by magnets, and released from it by cutting the electric current that feeds them. The piezoelectric load cell from the impactor was chosen for being accurate and for providing a linear response until a 22 kN load at a 20 kHz frequency.

Extremely high energy impacts are not interesting to understand the mechanical behavior of gres because, surpassing a certain release height threshold, the specimens will always fail but with different damage rates proportional to the potential energy attributed to the impactor. On the other hand, shock events with low energy that barely infer any damage to the coupon are also negligible, once besides ceramics being fragile, their plastic regime might not even be reached. Hence, iterative trials have been made to define a height in which, despite fracture being a possibility, it might also not be reached, so the first crack inducing mechanisms can be studied in detail. Once the precise falling height was defined, 6 low-velocity impact tests were performed: 3 for regular and 3 for annealed gres specimens.

Given that the upper and lower surfaces of a tile fired within a kiln may have distinct mechanical resistances, all tests were conducted setting the upper surface of the fired tile up.

### 2.3. Numerical Simulation

The usage of numerical simulations to describe impact events on ceramics is an extremely useful resource given the practicality and ease with which the response of a determined material can be defined without the need of experimental testing. The first constitutive numerical model for brittle materials was developed in 1991 when Espinosa et al. [29] built a damage mechanism of multiple micro cracks in different planes in a way that the crack could be propagated to any direction. Later, Johnson and Holmquist developed the most used constitutive models nowadays for brittle materials, the so-called JH-1 [30] and JH-2 [31], where the latter is a more complete version of the first accounting for gradually decreasing strength and bulking as a function of damage.

The JH-2 model, adopted in the present work, is widely preferred in the literature for impact simulations on brittle materials inclusively for high-velocity impact ballistic studies [32,33,34,35,36,37], where alumina, boron nitride and silicon carbide predominant compositions are used in armor structures given that they convert a significant amount of kinetic energy by fracturing their own matrix. However, the effectiveness of such constitutive model comes along with the need to properly calibrate a significant number of its parameters to ensure accurate simulation outcomes. Thus, the ideal procedure to verify the impact behavior of a certain material composition should also include experimental tests, once the proportion of elements used to manufacture a same type of ceramic may vary significantly enough to change its mechanical characteristics.

As in any constitutive model, the objective of JH-2 is to properly reproduce the behavior of a certain material to a loading condition. Intending to represent the ceramics inherent damage mechanism that begins with small cracks and grows into coalescent micro cracks forming a fracture, a variable represents the average damage for each finite element, which increases along with the deformation of the material resulting in strength loss. This damage grows with each iteration where the new values are based on the history variables of deformation to define the updated damage and strength (T).

Initially, the model considers the material as elastic, where the Equation (1) defines the material deformation (μ), through which the pressure (P) can be defined for a time step “n” and pressure coefficients K_1_ (equivalent to the bulk modulus), K_2_ and K_3_ as shown in Equations (2) and (3), that describe the pressure in compressed and tensioned elements, respectively. The history variable dependence of the model can be seen on ΔP_n−1_, which stands for the bulking pressure of the material determined by the amount of accumulated damage as a function of the pressure in the previous time step.

μ = (ρ/ρ_0_) − 1 (1)

P = K_1_μ + K_2_μ^2^ + K_3_μ^3^ + ΔP_n−1_ (2)

P = K_1_μ (3)

When the material is tensioned, it elastically deforms until a determined stress value set by the user, when brittle failure succeeds. Instead, under compressive loading, the damage starts to accumulate after a certain stress threshold. The intact material strength is represented by Equation (4), the fractured strength by Equation (5) and the current strength by Equation (6), where A, B, C, M and N are material-related constants; D stands for the total damage; T is the maximum tensile strength; $\dot{\varepsilon }$ is the strain rate; and the superscript ‘*’ indicates a normalized stress by the equivalent stress at the Hugoniot elastic limit.

(4) $$\sigma_{i}*=A(P*+T*{)}^{N}\;(1+\mathrm{Cln}\dot{\varepsilon })$$

(5) $$\sigma_{f}*=B(P*{)}^{M}\;(1+\mathrm{Cln}\dot{\varepsilon })$$

σ* = σ_i_* − D (σ_i_* + σ_f_*) (6)

By its turn, the increment in damage (ΔD, Equation (7)) is expressed as a function of the current increment in plastic strain (${\Delta \varepsilon }_{p}$) defined through the current strength found in Equation (6) through the radial return method [38] and the plastic strain to fracture ($\varepsilon_{f}$) given for a constant pressure, described by Equation (8) which includes further material-dependent constants D_1_ and D_2_.

ΔD = Δε_p_/ε_f_ (7)

ε_f_ = D_1_ (P* + T*)^D2^ (8)

The energy loss (ΔU) as a function of the increasing bulking pressure is described in Equation (9), which is dependent on the stress and shear modulus (G) of the material (Equation (10)). Besides the initial bulking pressure which is null for an intact material, the pressure of the next time step is calculated through Equation (11), where β is also a material constant, related to the fraction of elastic energy loss converted to hydrostatic energy (generally set to 1).

ΔU = U (D) − U(D_n+1_) (9)

U(D) = σ/6G(10)

ΔP_n+1_ = −K_1_μ + [(K_1_μ + ΔP)^2^ + 2βK_1_ΔU]^1/2^ (11)

The material intrinsic parameters present on the model described above are hence dependent on the composition of the ceramic considered. A comprehensive literature review allowed to conclude that the JH-2 (used in [32,33,34,36,37,39,40]) model is widely preferred over JH-1 (used in [35,41,42]) due to the possibility to compute accumulated damage. Different software tools are used by the authors such as AUTODYN [32,35,39,41], ABAQUS [33,43] and LS-DYNA [31,34,36,37], demonstrating good outcomes independently of the software selected, since the material model implemented is the same. Table 1 describes the material variables found in the literature for JH-2 models, highlighting the discrepant properties found for alumina.

As shown in Table 1, studies on alumina ceramics are predominant in the literature, where other works such as the ones from Burgüer et al. [33] and Feli et al. [37] also figure; besides, there are further contributions on silicon carbide made by Quan et al. [41], Holmquist et al. [42] and Hazell et al. [35], which use the same properties cited in Table 1.

Therefore, having in sight that all materials described above (with exception of SiO_2_) are utilized for ballistic armor, it is important to highlight that all the quoted studies portray high velocity numerical simulations. This review shows that not only gres porcelain has yet to be studied through numerical simulations properly and adapted to the JH-2 model, as there are no related studies regarding low-velocity impact on porcelain ceramics (i.e., for instantaneous impact velocities under 10 m/s), reinforcing the innovative essence of the current work. Defining the values of the gres constitutive properties is also challenging for the fact that the reference values found in the literature regard high-velocity impact, which infer a distinct damage and energy propagation mechanism on the slave specimen to that from low-velocity shocks.

To constitute the numerical model, the calibration of material-inherent properties was made for gres. Naturally, once this porcelain its mostly composed by alumina and silica, the properties were initially assumed as intermediate to ceramics solely composed by each material, as shown in Table 1. However, the optimal values obtained were not a simple weighted average of them, mainly because some variables are used as exponents in the constitutive equations and due to the other components present on gres’ structure that make a difference even if in small percentages. The calibrated values are shown in Table 2, being appropriate for both regular and annealed specimens, where the behavior variation was caused by their particular mechanical properties, taken from the results of the bending tests.

**Figure 5:**
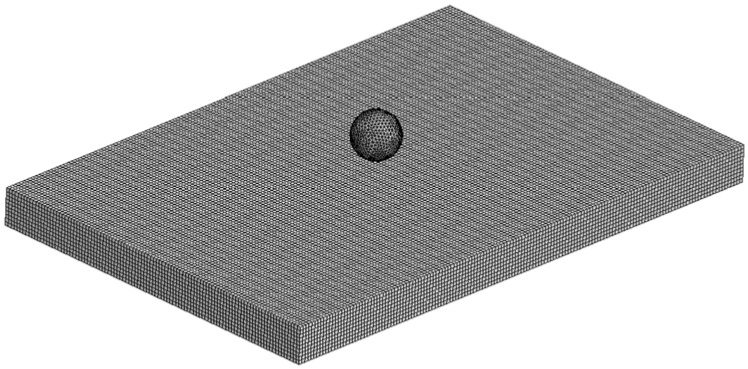
Finite element model representing the impact test.

Using the JH-2 implemented in LS-DYNA as MAT_110, a square mesh layout with 1 mm solid elements (Figure 5) was chosen for being able to provide a more accurate outcome for brittle materials given that it can compute more precisely the damage through all the extension of the model instead of only within a sphere of influence, which might be necessary in case of a large crack. Given the elevated fineness of the mesh, single integration point elements were used along with the Flanagan-Belytschko formulation [33,36]. For the contact definitions, AUTOMATIC_SURFACE_TO_SURFACE was preferred for yielding precise results in previous experiments and for surface-to-surface contacts being also used by other authors successfully [34,37]. Contrary to the majority of authors that usually use penalty formulation or soft constraint formulation, a pinball segment based contact was adopted because in the first cases the contact between indenter and slave was partial accounting for a certain level of physical overlapping between elements from different bodies. Instead, the selected option allowed the surfaces to be in full contact.

Furthermore, as advised by the literature [34,36,37], eroding definitions were adopted to avoid over-deformed and failed elements to interfere in the simulation generating a disturbance in the contact stress–strain data acquisition. A small time-step and 15 cycles between bucket sorts were used to ensure the stability and convergence of the simulations. The indenter was modeled as a rigid sphere with 12.7 mm in diameter, equivalent weight of 1.25 kg and non-deformable material with mechanical properties equivalent to steel through MAT_RIGID (or MAT_20).

## 3. Results

### 3.1. Flexural Tests

Bending test results are displayed in Table 3 for not annealed and in Table 4 for annealed specimens, where $F_{b}$ stands for breaking load, $\sigma_{b}$ for breaking stress, $\varepsilon_{b}$ for breaking strain and *E* for tensile modulus.

**Figure 6:**
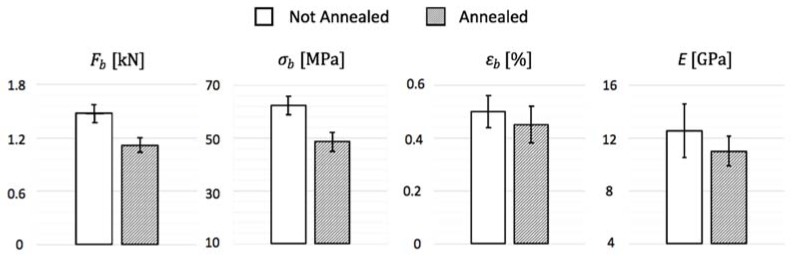
Comparison of bending test results.

**Figure 7:**
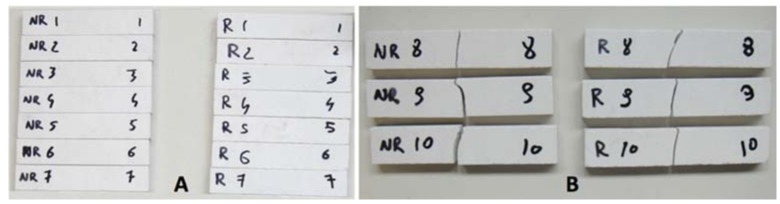
Annealed (R) and not annealed (NR) gres specimens before (**A**) and after (**B**) flexural tests.

Confronting the results obtained for the two types of porcelain tested in Figure 6, it is evident that the annealing process was not advantageous for the gres ceramic composition tested, given that the untreated porcelain presented higher maximum stress, maximum strain and elastic modulus, whereas the thermal cycle action made the material become more brittle and less resistant. Figure 7 shows pictures of the coupons before and after testing, highlighting that the specimens failed in their longitudinal center, offering precise results due to no influence of the support rods and the perfect alignment of the central rod. Unlike the fractures of the not annealed ceramics that present randomly oriented crack propagation directions, the unidirectional clean cracks in the annealed porcelain indicate sudden breakage, supporting the theory that the material has become more fragile after the thermal treatment given.

**Figure 8:**
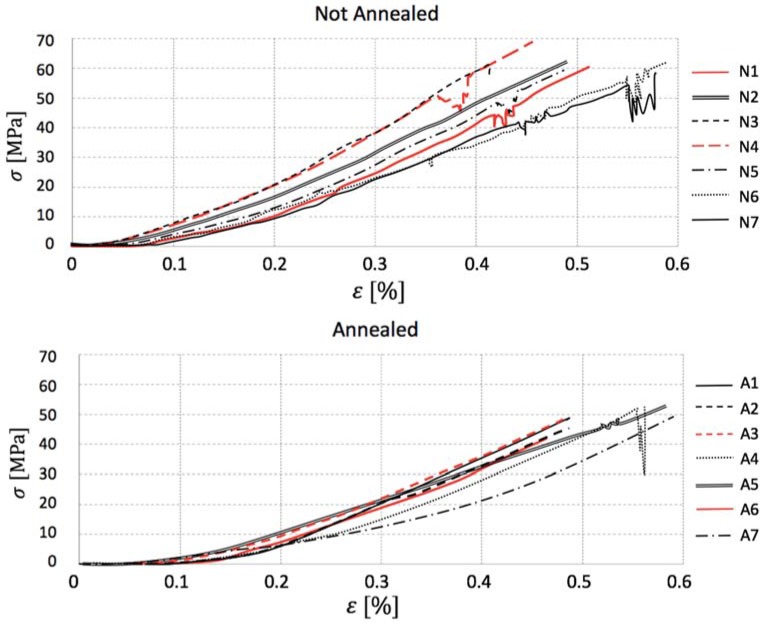
Comparison of bending test stress–strain curves.

The stress–strain loading curves shown in Figure 8 allow concluding a possible explanation for the loss in strength caused by the thermal treatment. Specimens that were not submitted to annealing are capable of resisting a certain level of damage without developing a terminal failure, as the stress decays on the loading curves show. In other words, even after developing small cracks, untreated gres is cohesive and tenacious enough to withstand higher stress levels without breaking. On the other hand, annealed porcelain succumbs at similar stress levels to those where the first cracks appeared on regular coupons not presenting any stress decays along all seven curves analyzed, meaning that the material becomes so fragile that it breaks when the microstructure discontinuities emerge. The premise is further validated by annealed specimen A4 that presented a stress decay and broke right after it.

### 3.2. Low-Velocity Impact Tests

The impact energy was determined by an iterative process that could define a height range for analyzing and comparing the tests among themselves in a way that the coupons were only partially damaged, instead of crushed. This range was comprised between 130 and 155 mm, corresponding to impact energies from 1.59 J to 1.90 J. A single reference test height was defined to facilitate the comparison of experimental data, being it 145 mm and causing the indenter to hit the slave with 1.78 J at a 1.69 m/s approximate instantaneous velocity.

**Figure 9:**
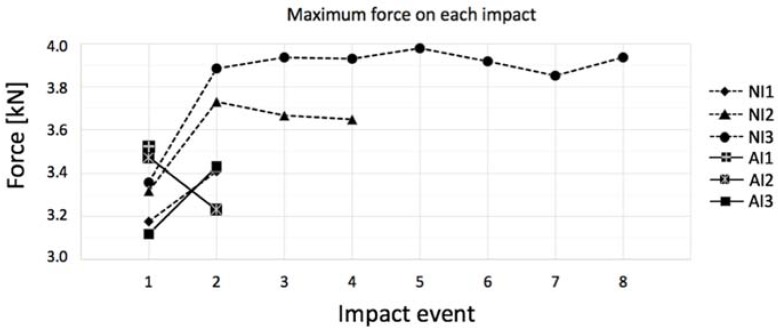
Maximum force in each successive impact event for not-annealed (NI) and annealed (AI) gres.

**Figure 10:**
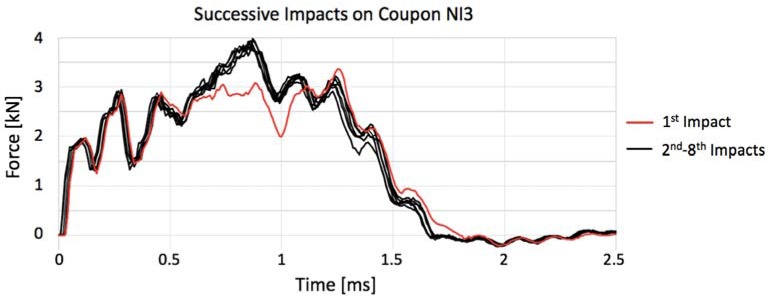
Impact curves of coupon NI3.

Because the selected impact energy was meant to be close to the gres’ resistance threshold, the manufacturing process of each specimen can account for small particularities, and the structure is not completely homogeneous and can have small internal defects as in any other ceramic, consecutive and equal impact tests were conducted in each specimen until terminal failure, where some specimens lasted longer than others. Table 5, which shows the number of impact events that each coupon was submitted, clarifies that the annealed porcelain becomes less resistant, breaking in two shocks as maximum, and the untreated stoneware not only has a higher average of impact to rupture, as it presents a coupon that lasted to eight impacts without breaking in the case of NI3. This behavior can be clearly seen in Figure 9. Moreover, NI (not-annealed) specimens show an enhancement in maximum contact force after the first impact event, as if the internal micro-cracks turned them into less ductile but still resistant structures. Since NI3, for instance, withstood eight consecutive impacts, as further impacts seemed unnecessary given that its maximum force was constant in all of them, similar to its behavior along all the force–time history curves shown in Figure 10.

**Figure 11:**
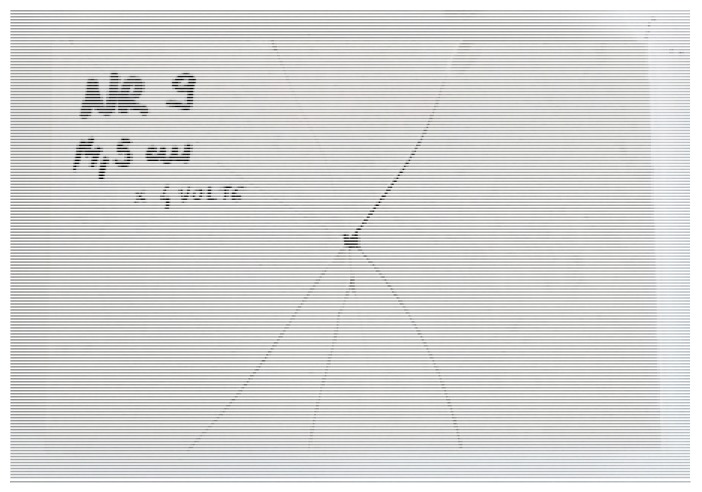
Example of specimen failure: NI2 after four impacts.

All specimens presented a certain level of damage after the first impact, resulting in harder and more fragile structures (NI1, NI2, NI3, and AI3) or coupons that would fail in the following (AI2) or even in the present shock (AI1) for being already too damaged internally (Figure 11). Hence, annealing gres has shown to be a disadvantageous thermal treatment in regards to its behavior against impact.

Matching classical low-velocity tests, the loading curves were represented with force–time data, given that defining stress would be unduly imprecise because the area of contact between the hemispherical tip impactor and the slave material varies over time depending on the penetration.

**Figure 12:**
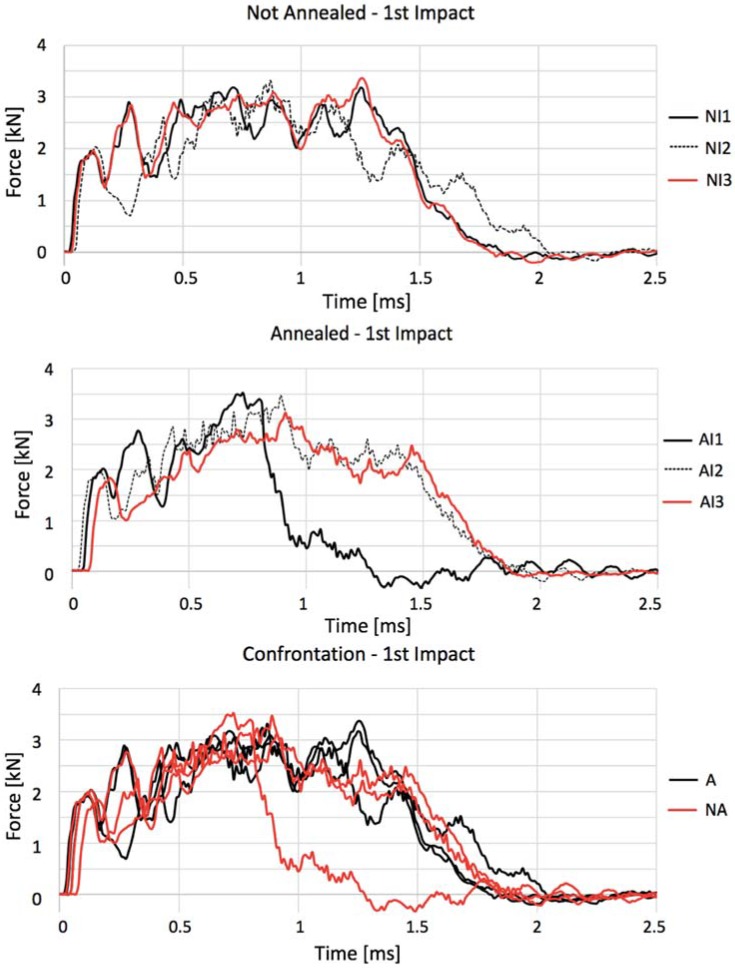
Force–time curves describing not annealed (NI1, NI2, and NI3) and annealed (AI1, AI2, and AI3) impacted specimens along with their comparison.

Once each impact is capable of inducing a certain amount of damage to the porcelain, Figure 12 shows the force–time history curves for the first impact in each intact coupon. Untreated gres again has a better impact absorbance for reaching its maximum force after a longer period than treated coupons, meaning that it is more ductile and has a higher damping performance. On the other hand, the response of all curves to the first fractures (from 0 to 0.75 ms) and to unloading (1.5 to 2.5 ms) is very similar. When annealed, gres depicts its peak force before 1 ms having also a more accentuated unloading slope. Regular gres, as an example of its higher ductility, absorbs the impact with higher cadency and more uniformly until 2 ms.

**Figure 13:**
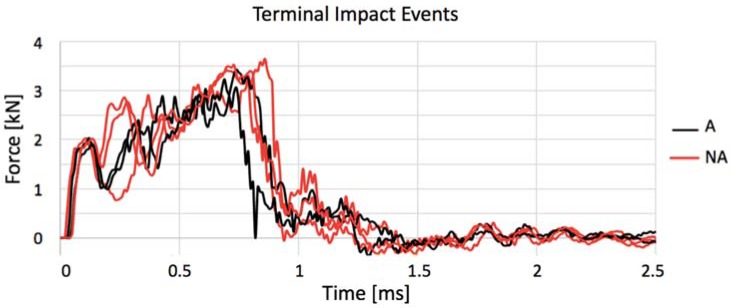
Comparison of terminal impact curves for annealed (A) and not annealed (NA) specimens.

In the terminal impact, all coupons present a behavior characterized by a high peak force followed by the unloading failure often before 1 ms (Figure 13, which does not regard NI3 because it did not fail). Among the annealed ones, AI1 showed this characteristic even in its first shock. Independently of how many impacts preceded failure, in all cases, the last shock curve is very similar, where the impact absorbance advantage feature of untreated gres has no influence anymore. These curves describe the emergence of failure through the formation of great fissures as a results of the coalescence of micro-cracks already existent.

**Figure 14:**
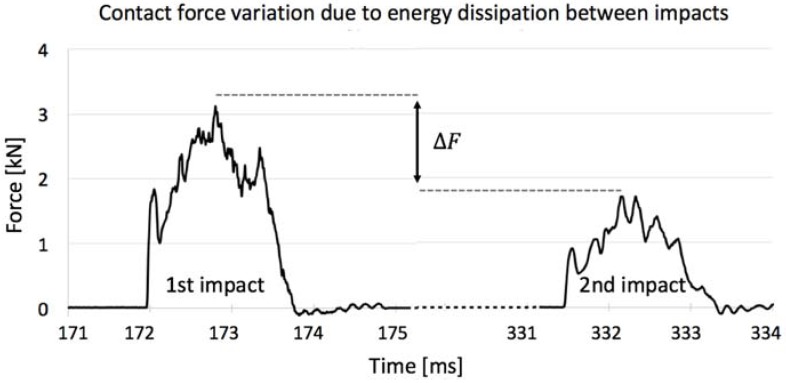
Contact force variation due to energy dissipation between impacts events.

Furthermore, aiming to determine the energy absorbed by the first impact, one sample of each kind was used in drop-weight tests in which the indenter was not stopped after the first contact generating a second impact, where in the latter the contact force had a smaller magnitude due to the energy that was dissipated in the first (Figure 14). In this way, through elementary physics, it is possible to determine the rebound height through the consecutive impact times when the force was maximum and the velocity of the indenter was null. Then, the difference in potential energy before the first and the second events can be calculated, representing the amount of energy that the ceramic absorbed disregarding the friction of air and the guiding columns of the testing machine, which were properly lubricated. Values of energy absorbed calculated were 21.5% in the annealed and 22.8% in the non-annealed porcelains, emphasizing the softer feature of untreated gres.

**Figure 15:**
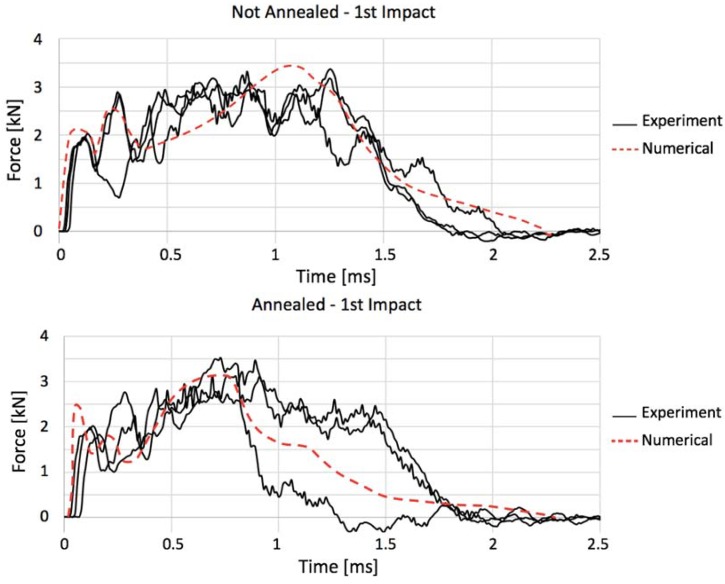
Confrontation of numerical and experimental curves for not annealed and annealed porcelain.

**Figure 16:**
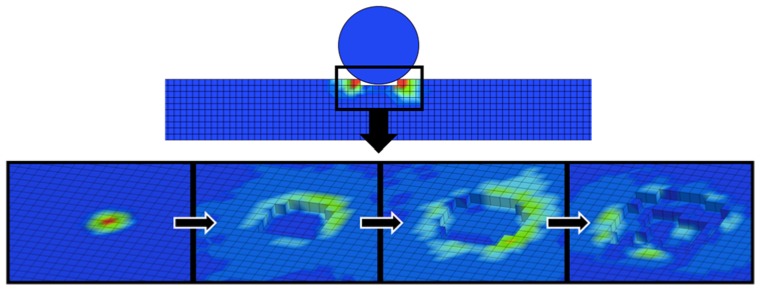
Evolution of damage caused by the impactor in the form of an indentation on a 1 mm square mesh showed through a von Mises stress gradient.

The numerical replication of the drop-weight experiment, shown in Figure 15, yielded according results, in a sense that the most prominent difference between the load–time curves for annealed and not annealed gres is that the first reaches its peak force before the latter. Furthermore, the impact period and maximum forces were on the same level for each case, conferring to the adjusted finite element model a sufficient level of accuracy to be able to reproduce faithfully this test. Besides the curve region near the peak force, where the most significant part of damage occurs making it difficult to be identically mirrored by finite elements, the regions regarding primary loading (0–0.25 ms) and unloading (1.5–2.5 ms) have been reproduced with noticeable precision in the not annealed case, whilst in the annealed one the unloading line was a middle-ground between the experiments that did not fail and the one that failed in the first shock. In none of the cases, the computational model presented terminal failure of the slave material; instead, the damage caused by the indenter was assimilated in the form of an indentation, given that it is not possible to simulate internal micro cracks within a finite element. The damage evolution is shown in Figure 16.

## 4. Conclusions

Analyzing gres porcelain ceramics in both regular and annealed forms, flexural and impact mechanical characterizations were successfully conducted through three-point bending and drop-weight low-velocity impact tests, the latter being afterwards suitably reproduced by a numerical model based on constitutive equations whose material-intrinsic properties have been properly calibrated for the studied composition.

Knowing that annealing is generally considered as a thermal treatment used to improve properties from materials due to the elimination of pre-existent manufacture-related internal stresses, the present work surprisingly showed that, for the case of the gres porcelain with constitution adopted, this treatment actually impairs its mechanical demeanor, turning the material into a more brittle and weak ceramic with reduced flexural strength and smaller impact damping capacity. This could be explained by the paradox that, even though internal stresses are diminished, the annealing continues the firing process, resulting in a possible mechanical resistance decrease. Furthermore, the standard deviation of bending and impact results can be partially attributed to the differentiated firing the tiles are submitted within an industrial kiln, where their position inside of it during firing influence their resulting properties.

Another explanation to this behavior can be found investigating the thermal cycle. For instance, the cooling rate after annealing of 12 °C/min might have been too high, creating more internal stresses than before, once ceramics have low thermal conductivity and the time necessary for the temperature gradient between the core and the surface of the tiles to be eliminated might be considerable. In this case, internal stresses would be created given that a compressive force would be exerted by the core that has a volumetric contraction limitation because of the already cooled, rigid and tensioned surface. This explanation is emphasized because dense ceramics must have a much slower cooling rate than porous ones, and gres accounts for a very dense variety of porcelain. However, on the thermal cycle, the maximum temperature the material was taken might have been too close to the state transition threshold, given that, for silica-predominant ceramics, the annealing temperature limit advised is 100 K below the glass-transition.

Despite profitable results obtained in which regards gres porcelain mechanical properties, further studies are advised for the characterization of its post-annealed form taking into account all the limitations regarding the particular thermal cycle that has to be carried as evidenced hereby.

## Figures and Tables

**Table 1:** Properties found in the literature.

Material	A	B	C	M	N	D_1_	D_2_	β	Source
Al_2_O_3_	0.88	0.28	0.007	0.60	0.64	0.01	0.7	1.0	Ong et al. [32]
	0.949	0.10	0.007	0.20	0.20	0.001	1.0	1.0	Tasdemirci et al. [34]
	0.989	0.77	0.0	1.0	0.376	0.01	1.0	1.0	Prakash et al. [39]
	0.93	0.31	0.0	0.60	0.60	0.005	1.0	1.0	Cronin et al. [40]
SiO_2_	0.93	0.088	0.003	0.35	0.77	0.053	0.85	1.0	Holmquist et al. [44]
SiC	0.96	0.35	0.0	1.0	0.65	0.48	0.48	1.0	Hallquist et al. [45]
B_4_C-SiC	0.94	0.65	0.005	0.85	0.67	0.001	0.50	1.0	Krishnan et al. [36]

**Table 2:** JH-2 calibrated material properties values for gres.

A	B	C	M	N	D_1_	D_2_
0.93	0.31	0.00	0.40	0.75	0.01	0.90

**Table 3:** Bending test results for not annealed gres porcelain.

	N1	N2	N3	N4	N5	N6	N7	
$F_{b}$ (kN)	1.45	1.44	1.48	1.67	1.44	1.46	1.40	1.48 ± 0.09
$\sigma_{b}$ (MPa)	61.18	62.13	62.83	69.57	60.26	61.61	58.47	62.29 ± 3.50
$\varepsilon_{b}$ (%)	0.50	0.48	0.42	0.45	0.49	0.58	0.57	0.50 ± 0.06
*E* (GPa)	12.02	12.70	15.08	15.31	12.17	10.51	10.15	12.56 ± 2.02

**Table 4:** Bending test results for annealed gres porcelain.

	A1	A2	A3	A4	A5	A6	A7	
$F_{b}$ (kN)	1.16	1.01	1.16	1.18	1.18	1.01	1.17	1.12 ± 0.08
$\sigma_{b}$ (MPa)	48.20	45.37	48.67	52.29	53.08	42.86	49.49	48.57 ± 3.60
$\varepsilon_{b}$ (%)	0.38	0.42	0.41	0.49	0.53	0.36	0.53	0.45 ± 0.07
*E* (GPa)	12.57	10.89	11.78	10.69	10.04	11.75	9.35	11.01 ± 1.11

**Table 5:** Number of impact events for rupture.

NI1	NI2	NI3	AI1	AI2	AI3
2	4	>8	1	2	2
